# Clinical Outcomes of 532 nm Long Pulse Laser Treatment for Facial Rosacea

**DOI:** 10.1111/jocd.70988

**Published:** 2026-06-17

**Authors:** Kian Hong Lau, Jin‐Hyun Kim, Jong‐Keun Song, Han Earl Lee, Hugues Cartier, Kyu‐Ho Yi

**Affiliations:** ^1^ EC Skin Laser Aesthetic Clinic Penang Malaysia; ^2^ You and I Clinic Seoul Korea; ^3^ Pixelab Plastic Surgery Clinic Seoul Korea; ^4^ Opening Plastic Surgery Clinic Seoul Korea; ^5^ Centre Médical Saint Jean Arras France

## 1 Introduction

Rosacea is a chronic inflammatory skin disorder characterized by facial erythema, telangiectasia, and, in some cases, papules and pustules. Granulomatous rosacea can also affect the nose, cheek, and chin, leading to phymatous rosacea [1, 2]. These manifestations can lead to significant psychosocial distress and a diminished quality of life for affected individuals [3]. While the exact etiology of rosacea remains unclear, factors such as vascular dysregulation, immune system anomalies, and environmental triggers are believed to contribute to its pathogenesis [4, 5].

Traditional management strategies for rosacea encompass topical and oral medications aimed at reducing inflammation and mitigating vascular symptoms. However, these treatments often yield variable results and may not adequately address the persistent erythema and telangiectasia associated with the condition. Consequently, laser and light‐based therapies have emerged as valuable adjuncts or alternatives in the therapeutic options against rosacea [6, 7, 8].

The 532‐nm wavelength potassium titanyl phosphate (KTP) laser has been investigated as a vascular‐targeting modality for superficial vascular lesions characteristic of rosacea. This wavelength closely aligns with the absorption peak of oxyhemoglobin, facilitating selective photothermolysis of dilated superficial blood vessels. Previous clinical studies have reported improvement in facial erythema and telangiectasia following KTP laser treatment, with generally favorable tolerability profiles under appropriate treatment parameters [9, 10, 11].

Despite the growing body of evidence describing the use of the 532‐nm KTP laser in rosacea treatment [9, 10, 11], data specifically examining its use in Asian clinical practice remain limited. Given the potential for variations in skin response due to differences in skin tone, ethnic background, and pigmentary risk, cautious evaluation of this treatment approach in Asian patients is warranted. This retrospective case series describes short‐ to mid‐term clinical observations following 532‐nm long‐pulsed laser treatment for facial rosacea in an Asian outpatient setting.

## 2 Materials and Methods

### 2.1 Study Design and Setting

This retrospective case series was conducted at EC Skin Laser Clinic, Penang, Malaysia, through a review of backdated medical records from December 2024 to May 2025. The study was based on routine clinical documentation rather than a prospectively designed interventional protocol. The study was conducted following the principles outlined in the Declaration of Helsinki. All patients had previously provided written informed consent for treatment and for the use of their anonymized clinical data and images for research and publication purposes.

### 2.2 Patient Selection

Medical records of patients who presented with and were clinically diagnosed with facial rosacea based on the updated 2019 rosacea diagnostic criteria [12], and who received treatment with a 532 nm long‐pulsed potassium titanyl phosphate (KTP) laser featuring a Variable Sequential Pulse structure and incorporated cryogen spray cooling (DermaV, Lutronic Co., South Korea), were reviewed. Cases were identified from available clinic records according to the predefined inclusion and exclusion criteria listed below. Because this was a retrospective review of available records, the possibility of selection bias cannot be excluded, particularly if cases with more complete photographic documentation and follow‐up were more likely to be included.

#### 2.2.1 Inclusion Criteria

- Age ≥ 18 years
- Clinical diagnosis of erythematotelangiectatic or mixed‐type rosacea
- Presence of persistent facial erythema and/or telangiectasia
- Completion of at least one laser session with available follow‐up data

#### 2.2.2 Exclusion Criteria

- Incomplete medical documentation
- Inadequate follow‐up

### 2.3 Outcome Assessment and Imaging Documentation

Clinical response was assessed retrospectively from serial clinical records and photographic documentation [13, 14]. Because this was a retrospective case series, standardized rosacea severity instruments such as the Clinician's Erythema Assessment (CEA), Investigator Global Assessment (IGA), or Patient Self‐Assessment (PSA) were not prospectively collected. Quantitative erythema measurements using objective imaging analysis or spectrophotometric indices were also not available. Therefore, outcome interpretation relied on serial clinical documentation and CPL‐assisted photographic comparison, which may introduce subjectivity and observer bias.

#### 2.3.1 Imaging Standardization

Images were obtained using the same Isemeco D9 3D skin analysis system across clinical visits when available. Device‐based imaging provided a more consistent platform for serial comparison than nonstandard clinical photography. However, because the study was retrospective and based on routine clinical images, complete standardization of all imaging variables—including facial expression, exact patient positioning, environmental conditions, and camera‐to‐face alignment—could not be fully verified. This limitation was considered when interpreting visual improvement.

#### 2.3.2 Adverse‐Event Monitoring

Adverse events were assessed retrospectively from clinical notes and follow‐up documentation. Events of interest included transient erythema, edema, blistering, purpura, pigmentary alteration, scarring, prolonged erythema, and paradoxical worsening. Because adverse‐event monitoring was not performed using a prospectively predefined safety scale, adverse‐event capture depended on clinician documentation and patient reporting during routine follow‐up.

#### 2.3.3 Adjunctive Medical Therapy and Supportive Care Documentation

Concomitant systemic and topical therapies were recorded as part of routine clinical care. Documentation regarding standardized counseling on sunscreen use, trigger avoidance, moisturization, barrier‐supportive skincare, and avoidance of irritating cosmetics was not consistently available in the retrospective records. Therefore, the potential influence of skincare optimization and flare‐trigger management on clinical outcomes could not be systematically assessed.

## 3 Results

A total of three cases met the inclusion and exclusion criteria. All patients were ethnic Chinese females with Fitzpatrick skin type III. Given the small sample size, retrospective design, and single‐center setting, these observations should be interpreted as descriptive and hypothesis‐generating rather than broadly generalizable. Follow‐up in this series was limited to 12 weeks, which captures short‐ to mid‐term clinical observation but does not allow assessment of longer‐term relapse patterns in this chronic relapsing condition.

### 3.1 Case 1

A 39‐year‐old ethnic Chinese female with Fitzpatrick skin type III presented with rosacea, characterized by fixed centrofacial erythema and inflammatory papules and pustules. She was treated with oral Roxithromycin 150 mg twice daily for 3 months, topical pimecrolimus 1% cream, and a series of 532 nm long‐pulsed KTP laser treatments (DermaV, Lutronic Co., South Korea). The laser treatment protocol is detailed in Table 1.

**TABLE 1:** Detailed laser treatment protocol used in case 1, including wavelength, fluence, VSP‐mode, pulse duration, spot size, pulse rate, and ICD Settings for DermaV; Lutronic Co., South Korea.

Parameters	Treatment 1		Treatment 2		Treatment 3	
Wavelength (nm)	532	532	532	532	532	532
Spot size (mm)	12	12	12	12	12	10
VSP mode	Single	Sub‐milli	Sub‐milli	Single	Sub‐micro	Single
Pulse width (ms)	10	10	10	0.3	10	0.3
Fluence (J/cm^2^)	4	5	5	0.8	4	0.8
Pulse rate (Hz)	Single	1	1	5	1	5
Intelligent cooling device (ICD) pre/delayed/post	10/10/10	15/15/10	15/15/10	Off	15/15/10	Off
Note	On papules and pustules	Over the rosacea area	Over the rosacea area	Full face	Over the rosacea area	Full face

**FIGURE 1:**
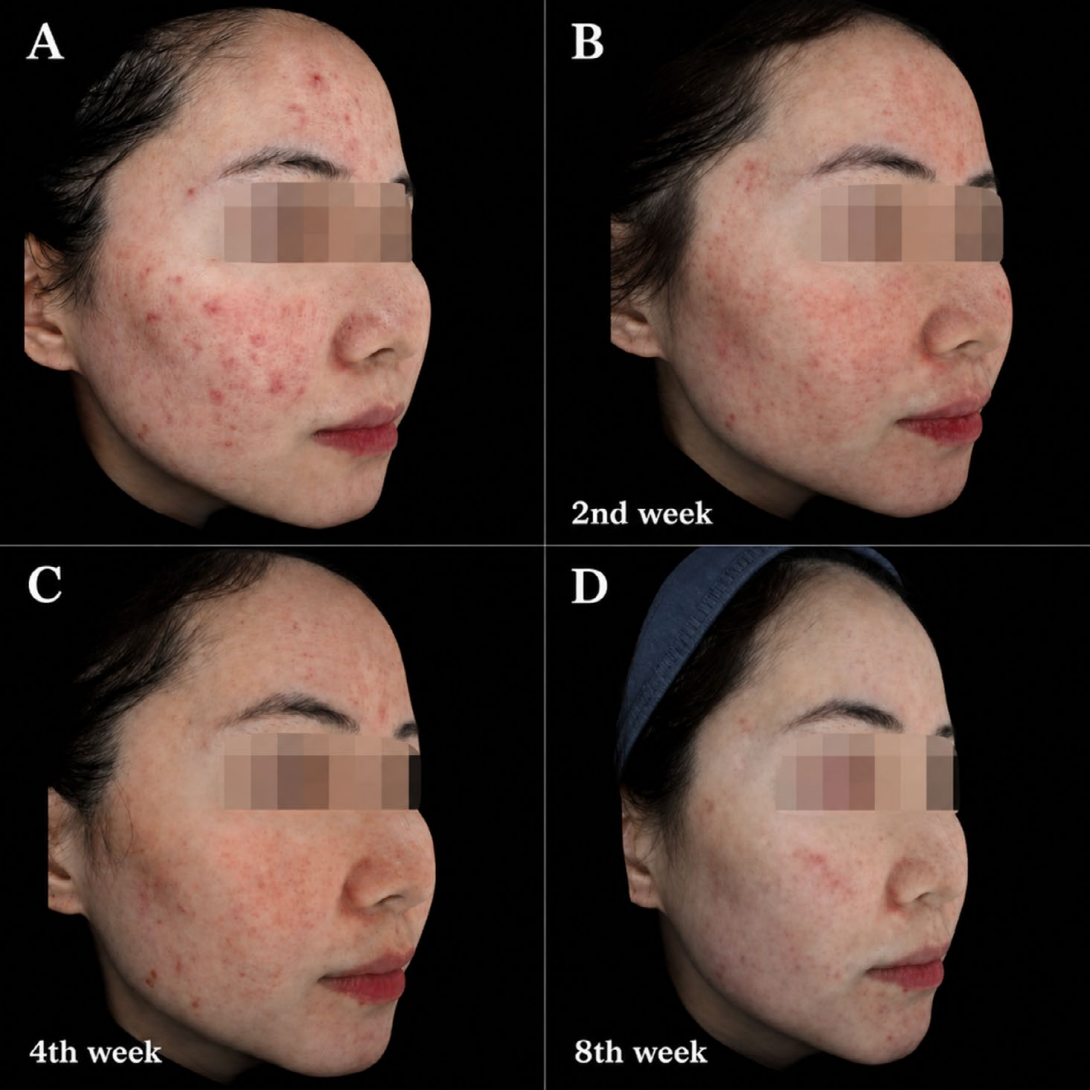
Digitally enhanced redness visualization images captured using the cross‐polarized light (CPL) mode, shown at baseline (A), 2 weeks after the first treatment (B), 2 weeks after the second treatment (C), and 4 weeks after the third treatment (D). Treatments were performed using a high‐energy, variable pulse duration, adjustable pulse structure 532 nm KTP laser equipped with integrated cryogen spray cooling.

Serial clinical and CPL‐assisted photographic documentation showed progressive visual improvement during the treatment course, with reduction in background erythema and inflammatory papules and pustules (Figure 1). Clinical photographs were captured using the 3D analysis system. Posttreatment effects were mild and self‐limiting, primarily consisting of transient erythema and swelling following each laser session. No blistering, purpura, pigmentary alteration, scarring, prolonged erythema, or paradoxical worsening was documented in the available clinical records.

### 3.2 Case 2

A 37‐year‐old ethnic Chinese female with Fitzpatrick skin type III presented with rosacea, predominantly characterized by fixed centrofacial inflammatory papules. She was treated with oral azithromycin 250 mg BD 3 times a week for 2 weeks, followed by roxithromycin 150 mg twice daily for 3 months, alongside topical pimecrolimus 1% cream, and underwent a total of five sessions of 532 nm long‐pulsed KTP laser therapy (DermaV; Lutronic Co., South Korea). The specific laser settings are summarized in Table 2.

**TABLE 2:** Detailed laser treatment protocol used in case 2, including wavelength, fluence, VSP‐mode, pulse duration, spot size, pulse rate, and ICD Settings for DermaV; Lutronic Co., South Korea.

Parameters	Treatment 1		Treatment 2		Treatment 3		Treatment 4		Treatment 5	
Wavelength (nm)	532	532	532	532	532	532	532	532	532	532
Spot size (mm)	12	12	12	12	12	8	12	8	12	10
VSP mode	Single	Sub‐milli	Single	Sub‐milli	Sub‐milli	Sub‐milli/sub‐micro	Sub‐micro	Sub‐milli/sub‐micro	Sub‐micro	Single
Pulse width (ms)	10	10	10	10	10	10	10	10	10	0.3
Fluence (J/cm^2^)	4	5	4	5	5	7–7.5	4	7.5–8	4	0.8
Pulse rate (Hz)	Single	1	Single	1	1	Single	1	Single	1	5
Intelligent cooling device (ICD) pre/delayed/post	10/10/10	15/15/10	10/10/10	15/15/10	15/15/10	15/15/10	15/15/10	15/15/10	15/15/10	Off
Note	On papules and pustules	Over the rosacea area	On papules and pustules	Over the rosacea area	Over the rosacea area	Visible vessel	Over the rosacea area	Visible vessel	Over the rosacea area	Full face

*Note:* Parameter adjustments were individualized based on the patient's clinical presentation, findings from digitally enhanced redness visualization images obtained using the 3Danalysis system, and real‐time laser endpoint observations.

Clinical progression was monitored using the 3D skin analysis system, with emphasis on the digitally enhanced redness visualization mode using cross‐polarized light (CPL) imaging.

**FIGURE 2:**
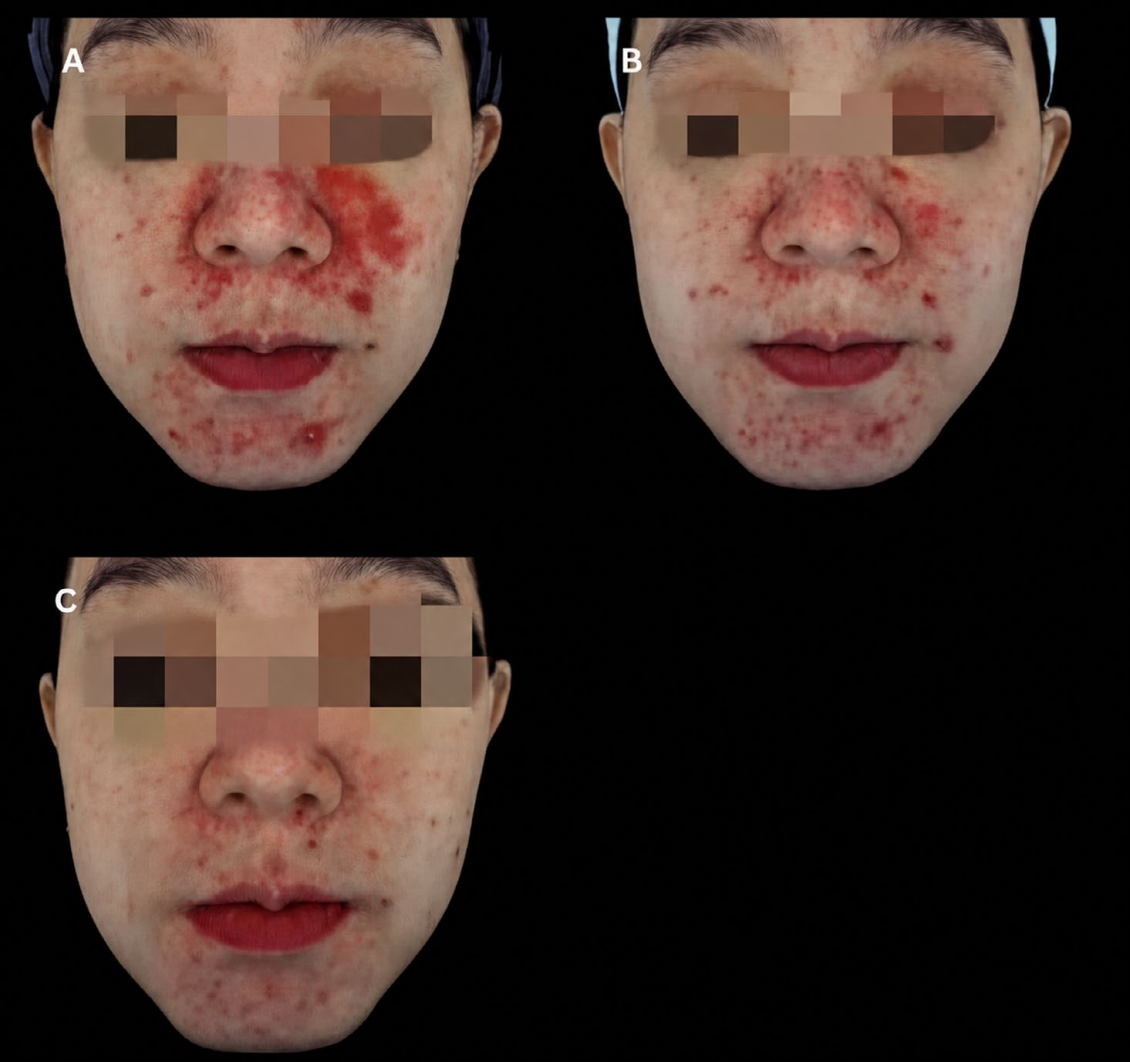
Digitally enhanced redness visualization images, showing (A) baseline, (B) 2 weeks after the first treatment, and (C) 4 weeks after completing a course of five laser treatments.

Serial clinical and CPL‐assisted photographic documentation showed visual improvement, with reduction in inflammatory lesions throughout the treatment course (Figure 2). The post‐laser recovery phase was limited to mild swelling and erythema, which resolved within 1–2 days. No blistering, purpura, pigmentary alteration, scarring, prolonged erythema, or paradoxical worsening was documented in the available clinical records.

### 3.3 Case 3

A 37‐year‐old postpartum ethnic Chinese female with Fitzpatrick skin type III presented with her first episode of rosacea, characterized by persistent centrofacial erythema and inflammatory papules. She was started on oral roxithromycin 150 mg twice daily, in combination with topical pimecrolimus 1% cream and topical clindamycin gel, targeting the inflammatory component.

In addition, the patient underwent four sessions of 532 nm long‐pulsed KTP laser therapy (DermaV; Lutronic Co., South Korea). The specific laser parameters used for each session are detailed in Table 3.

**TABLE 3:** Laser treatment parameters applied in Case 3, detailing the wavelength, fluence, VSP mode, pulse duration, spot size, pulse repetition rate, and integrated contact cooling (ICD) settings used with the DermaV system (Lutronic Co., South Korea).

Parameters	Treatment 1		Treatment 2		Treatment 3		Treatment 4	
Wavelength (nm)	532	532	532	532	532	532	532	532
Spot size (mm)	12	12	12	12	12	10	12	10
VSP mode	Single	Sub‐milli	Sub‐milli	Single	Sub‐micro	Single	Sub‐micro	Single
Pulse width (ms)	10	10	10	0.3	10	0.3	10	0.3
Fluence (J/cm^2^)	4	5	5	0.8	4	0.8	4	0.8
Pulse rate (Hz)	Single	1	1	5	1	5	1	5
Intelligent cooling device (ICD) pre/delayed/post	10/10/10	15/15/10	15/15/10	Off	15/15/10	Off	15/15/10	Off
Note	On papules and pustules	Over the rosacea area	Over the rosacea area	Full face	Over the rosacea area	Full face	Over the rosacea area	Full face

*Note:* Treatment settings were tailored for each session based on the patient's facial findings, vascular mapping obtained through digitally enhanced redness visualization using the 3D analysis system, and visual feedback from dynamic laser endpoint responses.

Laser treatment planning and parameter adjustment were guided by digitally enhanced redness visualization imaging.

**FIGURE 3:**
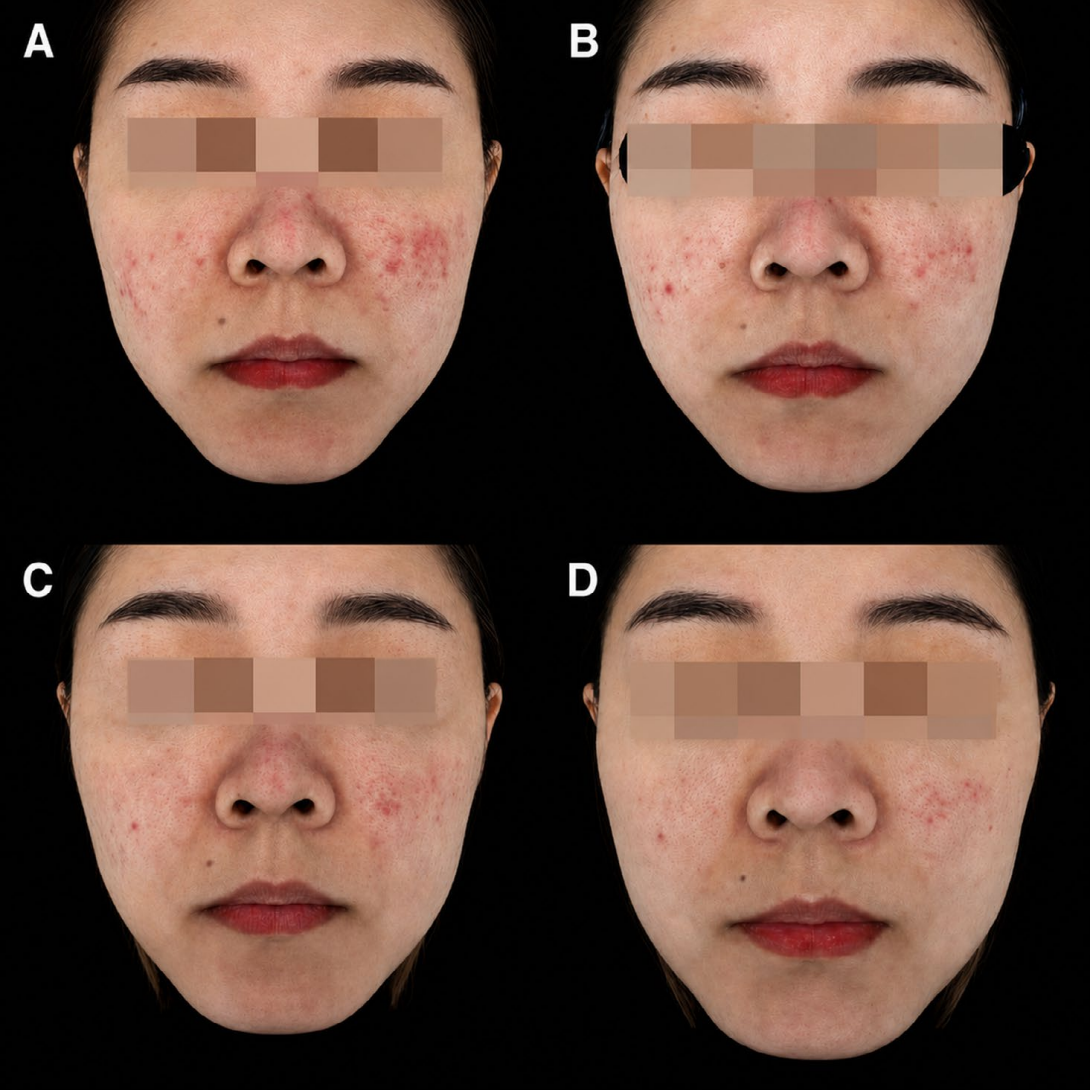
Digitally enhanced redness visualization images, showing (A) initial presentation, (B) improvement at week 3, (C) progress at week 6, and (D) follow‐up at week 12, 6 weeks after completing the fourth laser treatment.

Serial clinical and CPL‐assisted photographic documentation showed visible improvement as early as 1 week following the initial laser session, with progressive reduction in erythema and papular lesions noted during follow‐up (Figure 3). Post‐laser recovery was uneventful, with only mild, transient swelling that resolved within 1–2 days. No blistering, purpura, pigmentary alteration, scarring, prolonged erythema, or paradoxical worsening was documented in the available clinical records.

## 4 Discussion

Rosacea is a chronic inflammatory skin disorder with multifactorial pathophysiology. A growing body of evidence points to inflammatory angiogenesis, the abnormal formation of new blood vessels, as one of the central mechanisms in the disease process. This dysregulated vascular proliferation not only contributes to persistent facial erythema and the appearance of telangiectasia but also sustains inflammation by facilitating leukocyte trafficking and pro‐inflammatory cytokine release [15, 16]. Elevated levels of vascular endothelial growth factor (VEGF), cathelicidin LL‐37, and increased expression of matrix metalloproteinases (MMPs) have been documented in rosacea lesions, further supporting the link between vascular remodeling and disease severity [16, 17, 18]. Histological analyses have also demonstrated increased microvascular density (MVD) in lesional skin of rosacea patients compared to non‐lesional skin, which correlates with disease chronicity, clinical severity, and the presence of a dermal granulomatous reaction [19]. In this context, improvement in inflammatory papules observed alongside vascular features may be biologically plausible through secondary modulation of the vascular–inflammatory interface, including reduced vascular‐derived inflammatory signaling, cytokine‐mediated amplification, and neurovascular reactivity. However, this mechanistic interpretation remains hypothetical in the present case series and cannot be separated from the effects of concomitant anti‐inflammatory medical therapy.

Limitations and generalizability deserve emphasis. This report includes only three retrospectively identified patients from a single clinic, all of whom were ethnic Chinese with Fitzpatrick skin type III and predominantly erythematotelangiectatic or mixed inflammatory features. As such, the findings should be interpreted as a descriptive case series and cannot be used to infer efficacy for the broader spectrum of “Asian rosacea,” which is clinically heterogeneous and includes variable baseline severity, different phenotypes (e.g., predominantly papulopustular, granulomatous, or phymatous changes), and a wider range of skin phototypes (including III–V/VI) and ethnic backgrounds. While these cases may reflect a subset commonly encountered in East/Southeast Asian outpatient practice—persistent centrofacial erythema with inflammatory papules and visually appreciable vascular components—they do not represent all patient presentations or risk profiles. Larger, prospective, multiethnic cohorts are required to confirm reproducibility and to better define which Asian subgroups derive the most benefit with acceptable risk. Selection bias should also be considered. Because the cases were retrospectively identified from available clinic records, it cannot be determined from the present dataset whether all eligible patients treated during the study period were captured consecutively. Cases with more complete follow‐up, clearer photographic documentation, or more favorable visual outcomes may have been more likely to be included. This may overestimate the apparent clinical benefit and limits the interpretability of this small case series.

Traditional approaches to rosacea management, such as antibiotics or topical anti‐inflammatory agents, primarily target the inflammatory component but often fall short in addressing persistent vascular features such as erythema and telangiectasia [6, 7]. The application of vascular lasers, including 532 nm long‐pulsed KTP devices, may provide improvement in visible vascular components; however, in small non‐comparative series, conclusions regarding comparative advantage, independent treatment effect, or long‐term disease control should be made cautiously. By delivering targeted photothermal energy, these lasers selectively photocoagulate abnormal superficial vessels, potentially reducing microvascular density (MVD). This vessel clearance may help attenuate the angiogenesis–inflammation loop and support short‐ to mid‐term improvement.

Importantly, the observed clinical changes in this case series cannot be attributed solely to the 532 nm KTP laser. All three patients received concomitant systemic antibiotics and topical anti‐inflammatory agents during the treatment period, including oral roxithromycin and/or azithromycin, topical pimecrolimus, and topical clindamycin in one case. These medical therapies may have contributed to the reduction in inflammatory papules, background inflammation, and disease activity. Therefore, the laser component should be interpreted as part of a combined treatment strategy rather than as an isolated intervention. The relative contribution of laser therapy versus systemic and topical medical therapy cannot be determined from this retrospective, non‐comparative case series.

Because follow‐up in this series was limited to 12 weeks, durability beyond this period and any effect on recurrence cannot be determined.

Durability is particularly relevant in rosacea because disease activity can fluctuate with environmental exposures and seasonal triggers, and short‐term improvement may not translate into long‐term stability. Therefore, the present findings should be interpreted as short‐ to mid‐term observations rather than evidence of sustained remission. Future prospective studies should include longer follow‐up (≥ 24 weeks), predefined relapse criteria, and standardized clinician‐ and patient‐reported outcomes (e.g., erythema severity, flushing/burning symptoms, and quality‐of‐life measures) to better characterize time‐to‐flare and the durability of response after 532 nm VSP‐KTP treatment.

Another important aspect of this treatment protocol is the use of cross‐polarized light (CPL) imaging and digitally enhanced redness visualization via the Isemeco D9 3D skin analysis system. This noninvasive imaging approach can assist clinicians in assessing subsurface vascular patterns and in visualizing the distribution of redness beyond standard clinical inspection [13, 14]. By visualizing the microvascular network more clearly, CPL imaging may contribute to more targeted and informed laser planning [13, 14]. In the present retrospective series, however, CPL‐assisted images should be interpreted as supportive visual documentation rather than objective quantitative erythema measurements. Complete standardization of all photographic variables, including facial expression, exact positioning, environmental conditions, and camera‐to‐face alignment, could not be fully verified from the retrospective records.

To convert Tables 2 and 3 from a record of settings into a clinically interpretable guide, our parameter selection followed a phenotype‐ and vessel‐based logic: (i) vessel depth/caliber, (ii) inflammatory stage and background erythema, and (iii) treatment goal (diffuse redness‐reduction versus discrete telangiectasia clearance). In general, diffuse erythema with fine, superficial vascular networks was approached using shorter “submicro‐pulse” structures and conservative fluence with relatively longer effective heating windows to limit peak epidermal temperature while achieving gradual vessel heating. In contrast, larger and more defined telangiectatic vessels were treated using “submili” or single‐pulse structures with pulse durations selected to better match the thermal relaxation time of larger vessels, aiming for more efficient intravascular coagulation. Where background inflammation and papules were prominent, initial sessions emphasized global erythema reduction with lower‐to‐moderate energy delivery; once inflammatory activity and background redness improved, subsequent sessions could be focused more selectively on residual telangiectasia, using spot targeting and incremental adjustments as needed (Tables 2 and 3).

The use of oral macrolides in this series should be interpreted within the context of routine clinical practice rather than as a comparative systemic treatment strategy. Although tetracycline‐class agents, particularly doxycycline, are commonly discussed in rosacea management because of their anti‐inflammatory properties [6, 7], the patients in this retrospective series received roxithromycin and/or azithromycin as part of clinician‐directed medical therapy documented in the available records. This case series was not designed to compare macrolides with doxycycline or other systemic agents, and no conclusion can be drawn regarding the relative efficacy of different antibiotic classes.

Safety considerations, particularly post‐inflammatory hyperpigmentation (PIH), warrant deeper discussion in Asian skin where epidermal melanin competes for absorption at 532 nm. Shorter wavelengths can be highly effective for superficial vascular targets but may increase pigmentary risk in Fitzpatrick III–IV (and above) if epidermal thermal load is excessive. In this series (Fitzpatrick type III), no clinically significant pigmentary adverse events were documented, and posttreatment reactions were limited to transient erythema and swelling resolving within 1–2 days. Mechanistically, several elements of the platform and treatment strategy are relevant to PIH risk mitigation. First, the Variable Sequential Pulse (VSP) structure is designed to partition energy delivery over time rather than concentrating it into a single high‐peak pulse; this can reduce peak epidermal temperature while maintaining sufficient intravascular heating for selective photothermolysis. Second, integrated cryogen spray cooling provides rapid epidermal cooling immediately before/around energy delivery, helping to protect the epidermis and reduce nonspecific thermal diffusion. Third, we adopted a conservative, response‐guided escalation strategy—beginning with lower‐to‐moderate settings for diffuse erythema and adjusting gradually based on vascular mapping and real‐time endpoints—thereby minimizing overtreatment in melanin‐rich epidermis (Tables 2 and 3). From a practical standpoint, clinicians treating Asian patients with darker phototypes or higher pigmentary risk should consider a cautious, stepwise approach. This may include conservative initial fluence selection, test‐spot treatment when pigmentary risk is uncertain, gradual escalation only after assessing tissue response, adequate spacing between treatment sessions, and avoidance of treatment during active tanning, dermatitis, barrier disruption, or recent irritant reactions. Posttreatment photoprotection and avoidance of unnecessary irritation are also important to reduce the risk of post‐inflammatory pigmentary change.

Comparison with established light‐ and laser‐based modalities provides context for interpreting these observations. Pulsed dye laser (PDL; typically 585–595 nm) is widely used for rosacea‐associated erythema and superficial telangiectasia due to strong hemoglobin targeting and a robust evidence base; however, purpura and downtime may occur depending on pulse settings and patient factors. Long‐pulsed 1064 nm Nd:YAG offers deeper penetration and relatively lower epidermal melanin absorption than shorter wavelengths, which can be advantageous for deeper or larger‐caliber vessels and for patients at higher pigmentary risk, although it may be less efficient for very superficial diffuse erythema and can be more uncomfortable if aggressive settings are used. Intense pulsed light (IPL) is versatile for background erythema and dyschromia, but outcomes are device‐ and operator‐dependent, and in Asian skin careful parameter selection is required to minimize PIH risk.

Within this spectrum, a practical rationale for 532 nm VSP‐KTP in an Asian setting is strong targeting of superficial vascular components with strategies intended to reduce epidermal thermal load, including energy partitioning via VSP and epidermal protection via cryogen spray cooling. Nonetheless, because this is a small, retrospective, non‐comparative case series, these points should be viewed as mechanistic rationale rather than evidence of superiority. Future prospective head‐to‐head studies against PDL, IPL, and/or Nd:YAG using standardized outcomes and longer follow‐up are needed to define comparative effectiveness and safety.

Economic and practical accessibility should also be acknowledged. Advanced VSP‐KTP platforms equipped with cryogen spray cooling and CPL‐guided imaging may not be available in all clinical settings, particularly in resource‐limited practices. Equipment cost, operator experience, access to standardized imaging, and availability of integrated cooling may influence both treatment delivery and safety monitoring. Therefore, the protocol described in this series may not be directly generalizable to clinics without similar device capabilities, imaging support, or clinical experience.

Nonetheless, PIH risk cannot be assumed to be negligible across the broader Asian population, especially in darker phototypes (III–V/VI) or in patients with recent tanning, active dermatitis, or heightened post‐inflammatory pigment response. Future prospective studies should stratify outcomes by phototype, include standardized pigmentary assessments, and explicitly report PIH incidence and severity under predefined parameter ranges, cooling settings, and post‐care protocols to better quantify safety margins for 532 nm VSP‐KTP in diverse Asian subgroups.

Importantly, settings were not escalated by “numbers alone.” Instead, we applied a stepwise, response‐guided strategy: starting conservatively and adjusting fluence and/or pulse structure gradually based on (a) vascular mapping on CPL images, and (b) immediate clinical endpoints during treatment. Practical endpoints targeted a controlled vascular response, including transient vessel darkening/blanching and mild perifollicular edema, while avoiding excessive epidermal whitening or gray discoloration, prolonged purpura, or epidermal disruption. This approach provides a reproducible clinical rationale for why parameter ranges differed across sessions and patients, and why focal vessel‐targeting sessions used different pulse structures than global redness‐reduction sessions (Tables 2 and 3).

Several limitations should be emphasized when interpreting these findings. First, the study included only three retrospectively identified patients from a single center, without a control group. Second, standardized severity scores such as CEA, IGA, or PSA, as well as objective erythema quantification, were not prospectively collected. Third, all patients received concomitant systemic and topical medical therapy, preventing isolation of the independent contribution of laser treatment. Fourth, adverse‐event monitoring and skincare counseling were assessed from routine clinical documentation rather than predefined prospective protocols. Finally, follow‐up was limited to 12 weeks, preventing assessment of relapse, durability, or seasonal flare patterns.

## 5 Conclusion

In summary, this small retrospective case series provides descriptive, hypothesis‐generating observations on the use of a 532 nm long‐pulsed KTP laser with Variable Sequential Pulse structure and cryogen spray cooling in an Asian clinical setting involving ethnic Chinese patients with Fitzpatrick skin type III. Serial clinical and CPL‐assisted photographic documentation showed short‐ to mid‐term visual improvement with minimal documented downtime in the available records. However, because of the limited sample size, retrospective design, absence of standardized severity scoring, concomitant medical therapy, and 12‐week follow‐up duration, these findings should not be interpreted as evidence of validated therapeutic effectiveness or generalized to the broader Asian rosacea population. Future prospective studies with larger, multiethnic cohorts, broader phototype representation, standardized clinician‐ and patient‐reported outcomes, objective erythema quantification, predefined safety monitoring, and longer follow‐up are needed to clarify effectiveness, durability, relapse patterns, and pigmentary safety across diverse Asian subgroups.

## Author Contributions

Writing – original draft preparation: Kian Hong Lau and Jin‐Hyun Kim. Writing – review and editing: Kian Hong Lau and Jong‐Keun Song. Visualization: Kian Hong Lau, Han Earl Lee, and Hugues Cartier. Supervision: Kyu‐Ho Yi.

## Funding

The authors have nothing to report.

## Disclosure

Statement of human and animal rights: This retrospective case series was conducted in accordance with the Declaration of Helsinki and was based on a review of existing, de‐identified clinical records and images obtained in routine practice.

## Ethics Statement

This study involved human participants and was conducted in accordance with the ethical principles of the Declaration of Helsinki (2013 revision). All participants provided informed consent for participation and publication.

## Consent

All patients had previously provided written informed consent for treatment and for the use of their anonymized clinical data and images for research and publication purposes.

## Conflicts of Interest

The authors declare no conflicts of interest.

## Data Availability

The data that support the findings of this study are available from the corresponding author upon reasonable request.
